# Cap-dependent translational control of oncolytic measles virus infection in malignant mesothelioma

**DOI:** 10.18632/oncotarget.18656

**Published:** 2017-06-27

**Authors:** Blake A. Jacobson, Ahad A. Sadiq, Shaogeng Tang, Joe Jay-Dixon, Manish R. Patel, Jeremy Drees, Brent S. Sorenson, Stephen J. Russell, Robert A. Kratzke

**Affiliations:** ^1^ Department of Medicine, University of Minnesota, Minneapolis, MN, USA; ^2^ Department of Surgery, University of Minnesota, Minneapolis, MN, USA; ^3^ Department of Diagnostic and Biological Sciences, School of Dentistry, University of Minnesota, Minneapolis, MN, USA; ^4^ Department of Molecular Medicine, Mayo Clinic, Rochester, MN, USA; ^5^ Division of Hematology, Mayo Clinic, Rochester, MN, USA

**Keywords:** measles virus, oncolytic virus, translation, cap-dependent, mesothelioma

## Abstract

Malignant mesothelioma has a poor prognosis for which there remains an urgent need for successful treatment approaches. Infection with the Edmonston vaccine strain (MV-Edm) derivative of measles virus results in lysis of cancer cells and has been tested in clinical trials for numerous tumor types including mesothelioma. Many factors play a role in MV-Edm tumor cell selectivity and cytopathic activity while also sparing non-cancerous cells. The MV-Edm receptor CD46 (cluster of differentiation 46) was demonstrated to be significantly higher in mesothelioma cells than in control cells. In contrast, mesothelioma cells are not reliant upon the alternative MV-Edm receptor nectin-4 for entry. MV-Edm treatment of mesothelioma reduced cell viability and also invoked apoptotic cell death. Forced expression of eIF4E or translation stimulation following IGF-I (insulin-like growth factor 1) exposure strengthened the potency of measles virus oncolytic activity. It was also shown that repression of cap-dependent translation by treatment with agents [4EASO, 4EGI-1] that suppress host cell translation or by forcing cells to produce an activated repressor protein diminishes the strength of oncolytic viral efficacy.

## INTRODUCTION

Malignant mesothelioma (MM) arises from mesothelial cells in the pleura and is not curable with current therapies. The current standard of care for unresectable mesothelioma is combination chemotherapy of cisplatin and pemetrexed resulting in a median time to progression of 7 months and overall survival of 12 months. Overall response rates to this first line systemic therapy is only 41% in Phase 3 trials [1]. A promising approach for intracavitary cancers such as mesothelioma and ovarian cancer is viral oncolytic therapy. Studies using the Edmonston strain of the measles virus (MV) have been carried out or are underway in these diseases [2]. Previous data have demonstrated marked activity of the measles virus in xenograft models of mesothelioma with prolonged survival of murine models treated with oncolytic therapy [3]. These studies have led to a Phase 1 trial of measles virus therapy in pleural mesothelioma (ClinicalTrials.gov identifier NCT01503177).

CD46, expressed in all nucleated cells, is the primary receptor for Edmonston vaccine strains of measles virus. Wild-type MV strains typically interact with signaling lymphocytic-activation molecule (SLAM). Binding of MV hemagglutinin (MV-H) with CD46 triggers structural changes of MV-fusion protein (MV-F), necessary for syncytium formation, that leads to fusion of the viral envelope to the host cell membrane [4]. In normal tissues CD46 is expressed at low density while it is often overexpressed on many cancer tissues, resulting in a natural tropism for cancer cells while sparing normal cells [5]. Recently another receptor, nectin-4, has been identified for MV infection. In non-small cell lung cancer (NSCLC) nectin-4 has been associated with a poorer prognosis and is expressed in 58% of NSCLC tumors [6]. The incidence of nectin-4 expression in mesothelioma is unknown. Additionally, another factor important for viral tropism of cancer cells are deficiencies in the innate antiviral response pathway in cancer cells that elevates permissiveness for viral replication and spread selectively within tumors compared to normal cells [7].

In spite of these alterations there remain differential responses both *in vitro* and *in vivo* depending on the tumor model employed. Activation of cap-mediated translation in general results in translation of what appears to be a limited yet vital cohort of proteins associated with maintenance of the malignant phenotype [8]. Previous studies have shown 5′ cap-mediated translation of proteins is up-regulated in many or most cancers, including mesothelioma, and that downregulation of the eIF4F complex activity in mesothelioma is associated with loss of the malignant phenotype and increased sensitivity to cytotoxic therapies [9, 10]. In addition, viral infection in non-transformed cells is highly associated with redirection of cap-mediated translation away from production of proteins associated with host cellular maintenance and towards viral replication. Moreover, viruses are fully dependent on the host cell translation machinery to produce the proteins that are crucial for viral replication [11]. This is also likely true for viral infection of transformed cells. The hyper-activation of protein translation seen in the cancer phenotype may render transformed cells more sensitive to viral mediated oncolysis dependent upon the relative elevated levels of host cell protein synthesis.

In the current study, findings are presented which identify host cell cap-dependent translation as an important factor mediating measles virus activity against mesothelioma cells. Furthermore, results herein demonstrate that viral entry into mesothelioma cells is dependent upon the expression of CD46 and is independent of nectin-4.

## RESULTS

### Measles virus represses mesothelioma proliferation

**Figure 1:**
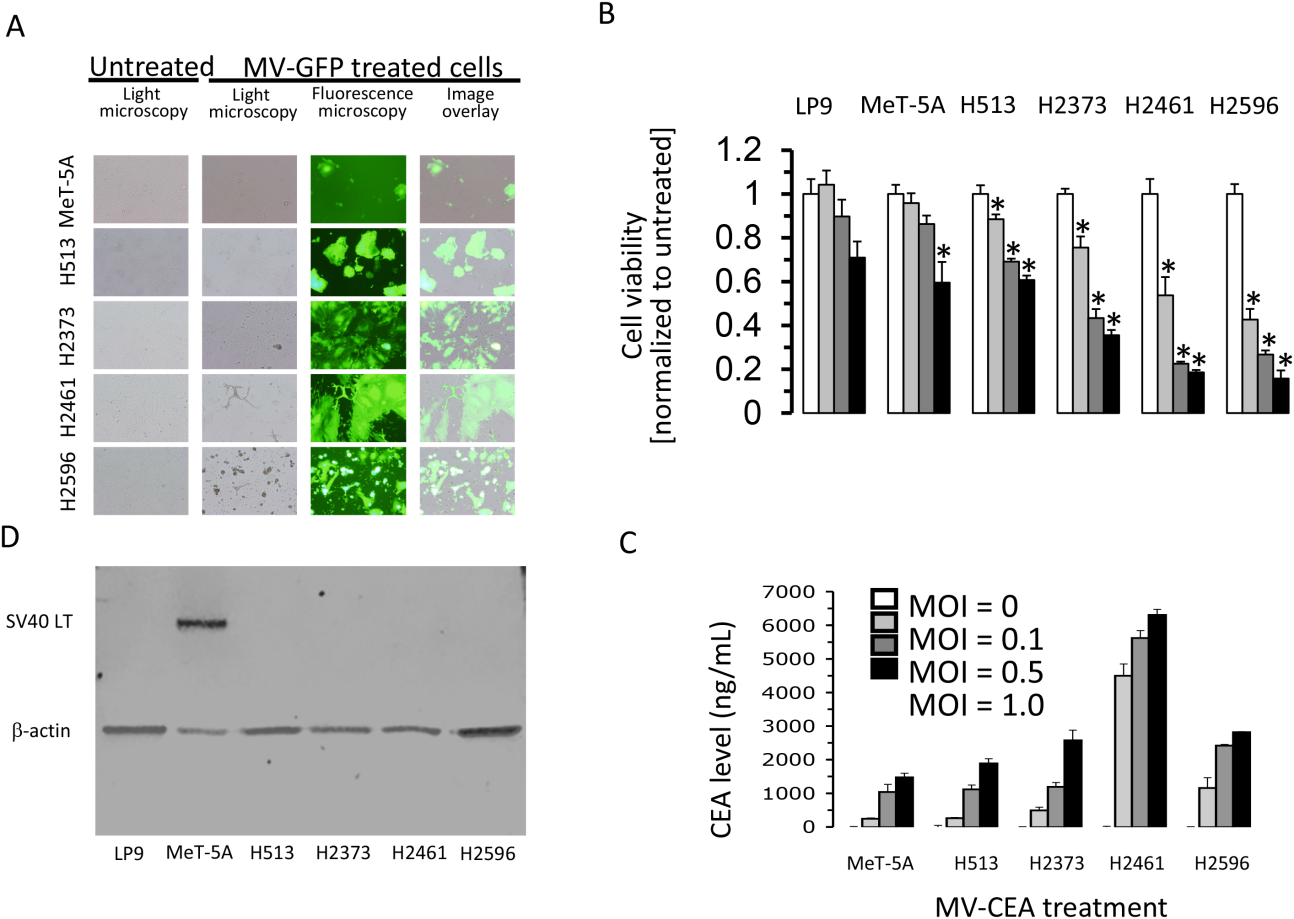
Treatment with measles virus inhibits proliferation of mesothelioma Immortalized mesothelial cells (MeT-5A) and MM cell lines (H513, H2373, H2461 and H2596) were treated with MV-GFP (MOI 1.0) and after 48 hours fluorescence and light micrographs (magnification 100X) acquired **(A)**. Human MM cells, immortalized mesothelial cells (MeT-5A) and mesothelial cells (LP9) were infected with MV-CEA at the indicated MOI and cell viability measured following 72 hours by trypan blue exclusion. Asterisks signify a significant difference in cell viability (*p*<0.05) between untreated cells and the same cells infected with MV-CEA **(B)**. Data represent the mean (+/− SD) of three independent measurements of cell number normalized to untreated cells. Production of human CEA was determined by collecting medium from MM and MeT-5A cells treated *in vitro* with MV-CEA at an MOI of 1.0 following 72 hours **(C)**. The presence of CEA was examined by ELISA technique. Error bars indicate standard deviation. LP9, MeT-5A and MM cell lysates from steady-state growth were prepared and immunoblots probed with SV40 large T antigen antibody. β-actin serves as a loading control **(D)**.

Previous research revealed that replication-competent measles virus strains can infect and inhibit growth of a wide variety of cancer types [2]. To test if mesothelioma cells are permissive to infection from the Edmonston vaccine strain (MV-Edm) of measles virus a panel of MM cell lines and a non-transformed cell line were treated with MV-GFP (green fluorescence protein producing measles virus) and fluorescence microscopy performed (Figure 1A). When compared to non-malignant immortalized mesothelial cells (MeT-5A) there is an increase in cytopathic effects as viewed by syncitia formation (Figure 1A) in characterized mesothelioma cell lines. The formation of syncitia (multinuclear aggregates) is characteristic of MV infection and denotes an efficient cell-to-cell spread of MV-Edm. To investigate the oncolytic strength of measles virus in mesothelioma, four MM cell lines were treated and assessed for cell survival and compared to non-transformed cells (Figure 1A). MM cells were treated with increasing multiplicities of infection (MOIs) of MV-CEA (carcinoembryonic antigen producing measles virus) for 72 hours and cells counted. In conjunction with increasing MOI of MV-CEA there is a stepwise decrease in cell viability that is more pronounced in mesothelioma cells, except for H513, than LP9 or MeT-5A cells (Figure 1B). Together these data show that at all MV doses cell viability is diminished substantially compared to untreated cells, and in three of four MM cell lines cell viability is decreased extensively, compared to non-transformed mesothelial cells. In addition, the CEA marker, a surrogate of viral gene expression, produced and secreted into the medium following cellular infection and replication of the measles virus, increases inversely with cell viability following exposure to increasing doses of MV-CEA (Figure 1C). To verify the MeT-5A cell line immunoblot analysis was employed determining that the SV40 large T antigen is produced. The generation of MeT-5A started by employing healthy human mesothelial cells that were infected with plasmid pRSV-T (an SV40 ori - construct containing the SV40 early region of the Rous sarcoma virus long terminal repeat) that lead to creation of the immortalized cell line [12]. These cells, in the original work, produced the SV40 large T antigen, as do the MeT-5A cells used in the experiments presented in this investigation (Figure 1D).

### Apoptosis is induced by measles virus in mesothelioma

**Figure 2:**
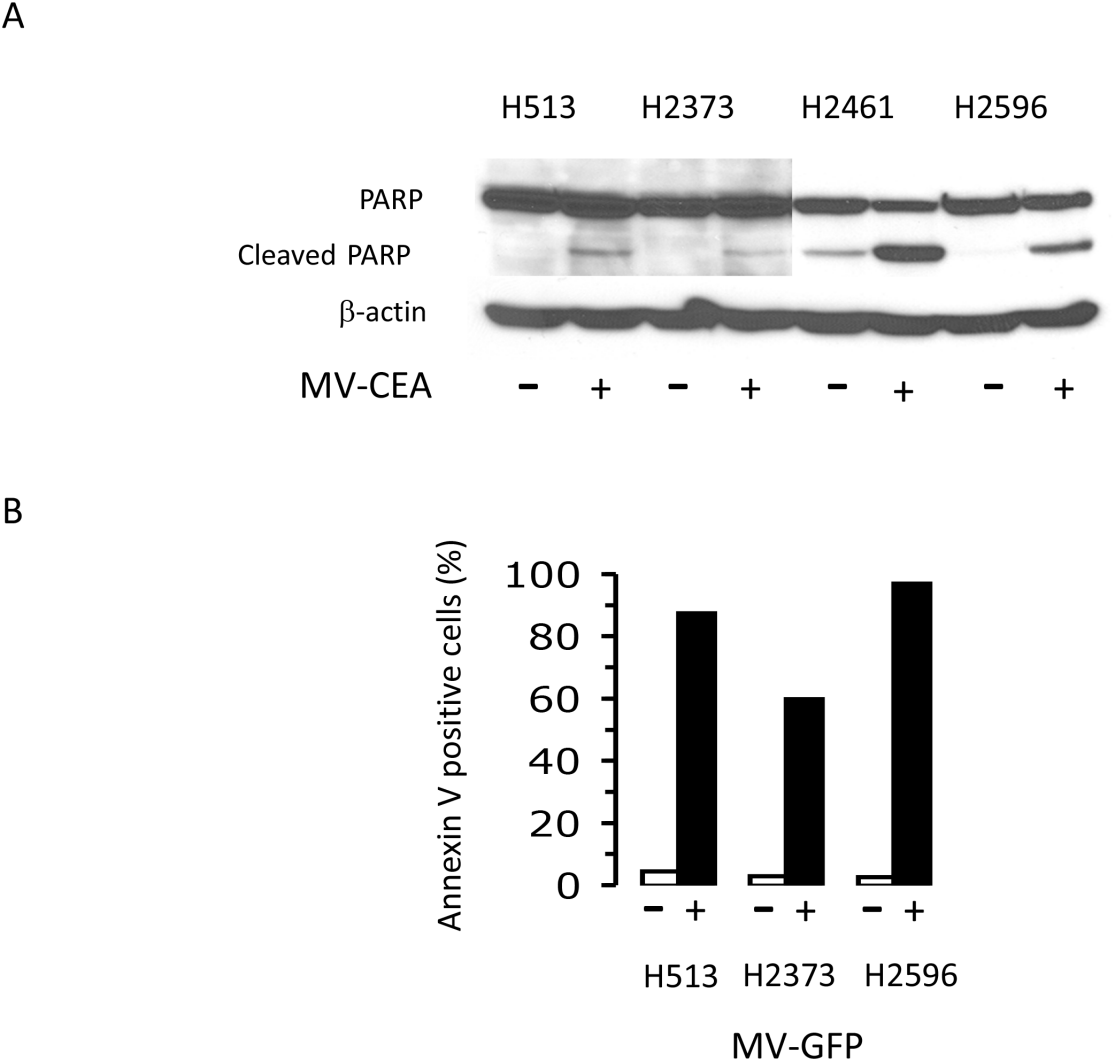
Measles virus treatment induces apoptosis in mesothelioma Mesothelioma cells were untreated or treated with MV-CEA with an MOI of 1.0 for 48 hours. Lysates were prepared and immunoblots probed with anti-PARP antibody. β-actin serves as a loading control **(A)**. Flow cytometry analysis results of H513, H2373, and H2596 for Annexin-V staining are assessed in mesothelioma cells treated and not treated with MV-GFP (MOI 1.0; 48 hours) **(B)**.

Two markers of apoptosis were studied that include poly (ADP-ribose) polymerase (PARP) cleavage, and Annexin V staining. PARP cleavage was assessed in MM cells treated with MV-CEA as an indicator of apoptosis induction. The panel of 4 MM cell lines were either treated with MV-CEA (MOI of 1.0) or left untreated for 48 hours and lysates prepared. Immunoblot analysis revealed that MV-CEA treatment led to increased PARP cleavage denoting apoptosis as compared to untreated cells (Figure 2A). A consequence of apoptosis that can be measured by Annexin V staining is the externalization of phosphatidylserine. Three MM cell lines were either treated with MV-GFP (MOI of 1.0) or left untreated for 48 hours and cells were subjected to Annexin V measurement (Figure 2B). The increase in the level of Annexin V-positive cells was from 4.4 to 87.4% for H513 cells, from 2.96 to 59.9% for H2373 cells and from 2.6 to 97% for H2596 cells following MV-GFP treatment. Results from both of these different apoptosis assays demonstrate that measles virus is capable of invoking apoptotic cell death in mesothelioma cells.

### CD46 levels are elevated in mesothelioma

**Figure 3:**
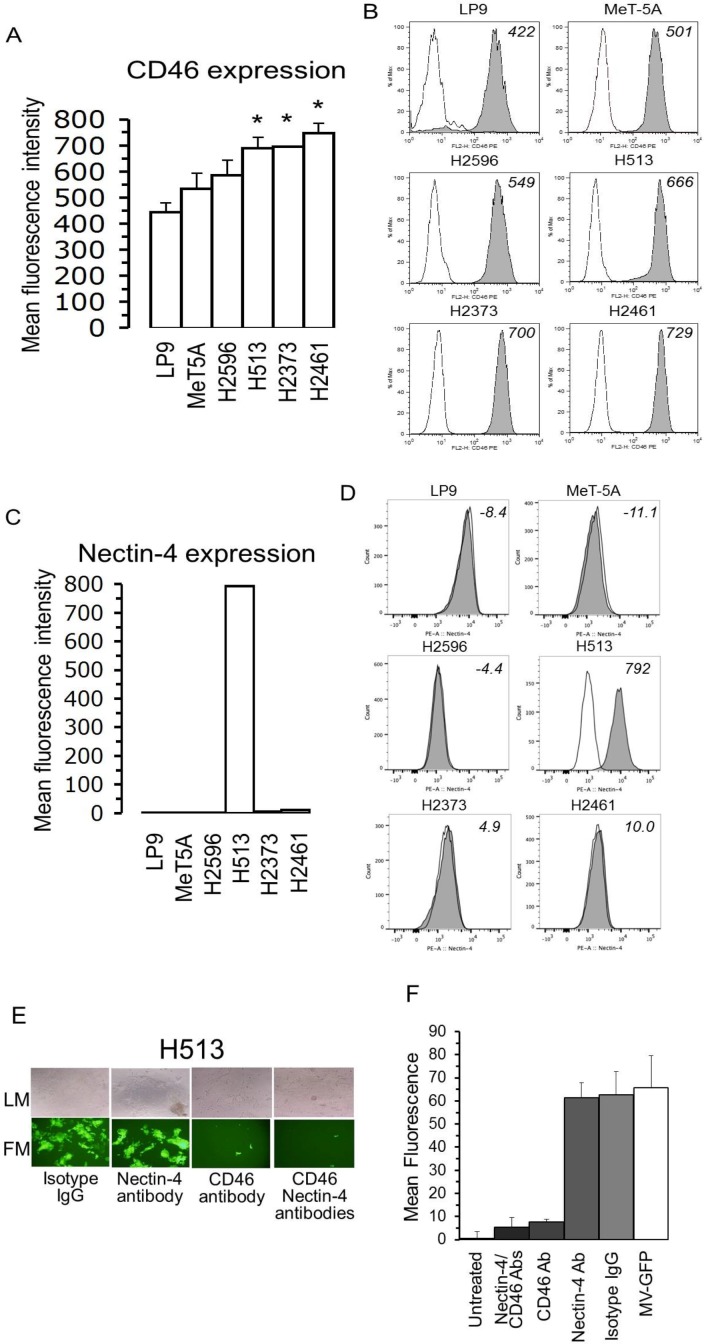
In mesothelioma CD46 levels are elevated and host cell entry is reliant on CD46 receptor and not nectin-4 Relative CD46 expression levels on surfaces of a panel of non-transformed and MM cells expressed in mean fluorescence intensity **(A)**. Representative histograms of CD46 receptor are displayed from non-transformed and mesothelioma cells. Cells were stained with anti-CD46 PE antibody (grey histogram) or an isotype control (open histogram) and analyzed by flow cytometry **(B)**. Nectin-4 expression levels on the surfaces of non-transformed and MM cells conveyed in mean fluorescence intensity **(C)**. Histograms of nectin-4 on cells stained with anti-nectin-4 PE antibody (grey histogram) or an isotype control (open histogram) and measured by flow cytometry **(D)**. Microscopy, both Light (LM-top) and fluorescence (FM-bottom) of H513 cells infected with MV-Edm producing green fluorescent protein (MV-GFP) following exposure to blocking antibodies against CD46, nectin-4 or the combination **(E)**. Results for fluorescence measurement following H513 cell treatment with blocking antibodies for CD46 and nectin-4 prior to MV-GFP infection. Isotype IgG treatment and H513 cells untreated with MV-GFP were used as positive and negative controls, respectively **(F)**.

Previous studies demonstrated that the cytopathic effects in tumor cells resulting from MV-Edm infection, is dependent upon a threshold CD46 receptor density [5]. The critical component of this phenomenon is that below the threshold CD46 density virus-induced cell-to-cell fusion occurs at a minimal level but above the threshold CD46 level cell-to-cell fusion increases dramatically resulting in amplified cytopathic effects. The level of MV-Edm receptor, CD46, in MM cells was measured and compared to non-transformed and non-immortalized (LP9) and non-transformed but immortalized (MeT-5A) cells (Figure 3A). The CD46 receptor level on three of four MM cell lines was significantly higher than in the LP9 control cells (Figure 3A, 3B). The level of CD46 may partly explain the oncolytic specificity of MV-Edm for MM cells displayed in Figure 1. However, based on CD46 receptor level alone it would be expected that H513 would be more sensitive and H2596 less sensitive to measles virus than what was revealed in the cell viability studies.

### MV-Edm relies on CD46 receptor and not nectin-4 for MM host cell entry

For both the wild-type and vaccine strains of MV, nectin-4 was recently identified as a receptor [13, 14]. To assess the relevance of nectin-4 as a receptor for MV-Edm in mesothelioma infection the level of nectin-4 was determined (Figure 3C and 3D). A panel of 4 MM cell lines, along with non-transformed mesothelial cells, was analyzed for nectin-4 expression employing flow cytometry. Nectin-4 was highly expressed only in H513 cells, the MM cell line least sensitive to MV-Edm. To explore the reliance of MV-Edm on nectin-4 and CD46 receptors for H513 host cell entry, cells were treated with blocking antibodies against nectin-4, CD46 or both. As a control isotype IgG antibodies were used. Following antibody treatment H513 cells were treated with MV-GFP (MOI 1.0) and analyzed by fluorescence microscopy (Figure 3E). Fluorescence of treated cells was measured employing a plate reader (Figure 3F). The viral infection of H513 was not impacted by the addition of blocking antibodies to nectin-4. However, the blocking CD46 antibody attenuated, nearly-completely, infection by MV-GFP. Blocking both receptors with antibodies also halted MV-GFP infection. The fluorescence measurement following antibody treatment corroborates these results. Exposure to nectin-4 blocking antibody was permissive for MV-GFP infection (high mean fluorescence) while the exposure to CD46 blocking antibody (low mean fluorescence) was not. These results suggest that for H513 cells that express nectin-4, MV-GFP viral entry depends upon CD46 and not nectin-4. In summary, three of four MM cell lines did not produce nectin-4 while the cell line that did was not reliant upon nectin-4 for viral entry but depends on CD46 (Figure 3C-3F).

### Host cell translation inhibition suppresses measles virus potency

**Figure 4:**
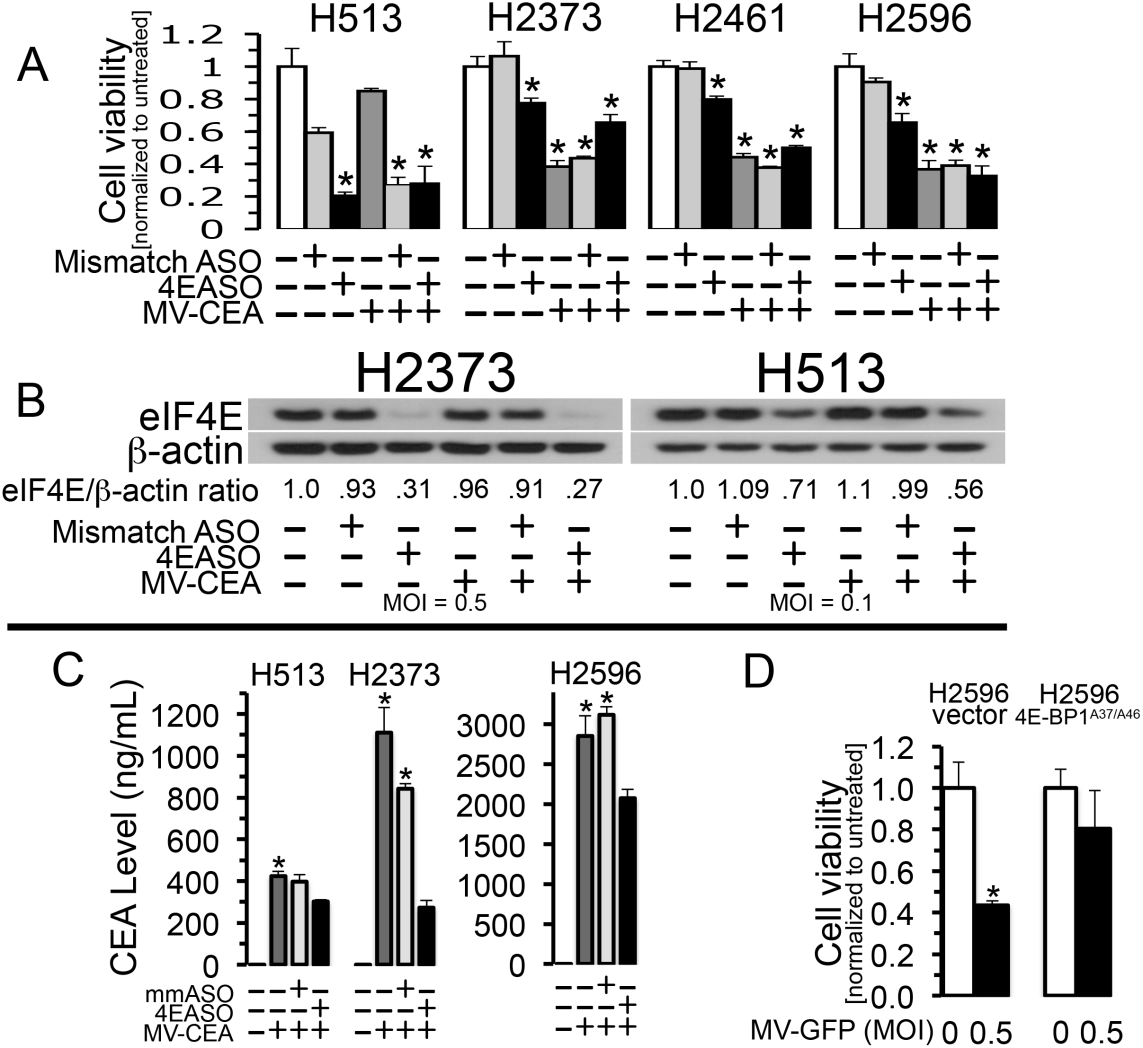
Measles virus potency is reduced during inhibition of host cell translation Mesothelioma cells were transfected with mismatch antisense oligonucleotide (mmASO) or eIF4E-specific ASO (4EASO) and were or were not treated with MV-CEA. H513, H2461 and H2596 were treated with MOI of 0.1 and H2373 was treated with MOI of 0.5, where indicated. H513, H2596, H2461 and H2373 were treated with 200 nM, 50 nM, 100 nM and 100 nM ASO, respectively. Following 72 hours treatment viable cells were counted. Asterisks denote a significant difference in cell viability (*p*<0.05) between untreated cells and the same cells treated with MV-CEA, mmASO, 4EASO or the combination treatment. **(A)**. Mesothelioma cell lines H2373 and H513 were untreated, treated with MV-CEA at the signified MOIs or transfected with mmASO or 4EASO (200 nM for H513 and 100 nM for H2373) or the combination of agents for 48 hours. Lysates were prepared and immunoblots probed with anti-eIF4E antibody. β-actin serves as a loading control. The normalized ratio of eIF4E to β-actin after treatment is shown below the immunoblot. ImageJ, a public domain java image processing program was used to measure protein band intensity. **(B)**. Human CEA production was determined by collecting medium from MM cells untreated or treated *in vitro* with MV-CEA, mmASO plus MV-CEA or 4EASO plus MV-CEA as in (4A). The presence of CEA was examined by ELISA technique. Standard deviation is represented by error bars. Denoted by asterisk is the significant difference in CEA levels between combination treatment of 4EASO plus MV-CEA compared to the combination treatment of mmASO plus MV-CEA or MV-CEA alone (*p*<0.03) **(C)**. H2596 either expressing activated 4E-BP1^A37/A46^ or not were treated with the indicated MOI of MV-GFP for 72 hours and viable cells counted. Averages were compared for H2596 empty vector between treated and untreated cells by Student's *t*-test. * denotes a p value equal to 0.012. **(D)**. Cell viability is expressed as cell number normalized to untreated cells. Error bars indicate standard deviation of the mean.

One of the primary toxic effects of oncolytic viral infection of cancer cells is redirection of cap-mediated protein translation away from necessary host cellular metabolism and towards viral replication. Indeed, viruses are potently dependent on the translation machinery of their host cells for viral protein production. These proteins are required for viral genome replication and progeny virion production [11]. With this in mind, down regulation of eukaryotic initiation factor 4E (eIF4E), the rate limiting protein of the eukaryotic initiation complex (eIF4F) should blunt viral replication and cytotoxicity engendered by measles virus. To explore the impact that inhibition of cap-mediated translation has on the oncolytic activity of MV-Edm for mesothelioma, cells were treated with a translation inhibitor, measles virus or both agents. An eIF4E targeted antisense oligonucleotide (4EASO) previously demonstrated to diminish eIF4E protein levels was used to attenuate cap-mediated translation. A mismatch control ASO (mmASO) was employed for comparison [15]. mmASO had an inhibitory effect upon cell line H513 and this effect may be specific for this cell line but did not impact the viability of the other cell lines studied. When mesothelioma cells are transfected with 4EASO there is an expected decrease in eIF4E protein levels, demonstrated with immunoblot analysis, and concordant decrease in cell viability when compared to mmASO (Figure 4A, 4B). Cell viability was not reduced in three of four cell lines treated with the combination of MV and 4EASO compared to at least one of the single agent treatments alone. However, for H2596 the diminishment between MV and the combined treatment was slight compared to MV-CEA alone (Figure 4A). To verify that the reduced cytotoxicity from inhibited host cell cap-dependent translation is due to decreased viral production, CEA levels in culture medium from MV-CEA infected and ASO treated cells was measured. Previous work indicated that an increase in MV-CEA titer was directly associated with an increase in the CEA level in culture medium that correlates with viral replication and viral gene expression [16]. CEA marker secreted into the medium following MV-CEA infection of cell lines H513, H2373 and H2596 decreased significantly with the addition of 4EASO to the MV-CEA infected cells (Figure 4C). This suggests that during inhibition of host cell translation MV-CEA production is reduced. These results together indicate that in the presence of limiting and diminished levels of eIF4E the mesothelioma cells are rendered less sensitive to oncolytic viral effects of MV-CEA.

Further, if repressing cap-mediated translation by reducing eIF4E levels attenuates measles virus cytotoxicity, then forcing mesothelioma cells to produce an endogenous repressor protein of eIF4E, 4E-BP1, should also result in a similar decreased sensitivity to measles virus oncolysis. Mesothelioma cells were selected to ectopically express a dominant active form of 4E-BP1 with residues threonine 37 and 46 replaced with alanines (4E-BP1^A37/A46^), rendering the resulting protein insensitive to phosphorylation and thus constitutively active in its repressor function. In previous studies, mesothelioma cells producing 4E-BP1^A37/A46^ were shown to have strongly diminished assembly of the eIF4F cap-dependent translation complex compared to cells not producing 4E-BP1^A37/A46^ [10]. To evaluate the ability of 4E-BP1^A37/A46^ to repress measles virus cytopathic activity MM cell line H2596 was stably transfected with empty vector or with the gene for dominant active 4E-BP1^A37/A46^. These cells were then either left untreated or treated with MV-GFP at an MOI of 0.5 (Figure 4D). Cell viability following MV-GFP treatment led to reduced cytotoxicity in cells producing dominant active 4E-BP1^A37/A46^ compared to the same cells not producing 4E-BP1^A37/A46^. The results promote the notion that host cell translation potency controls oncolytic MV efficacy.

Additionally, based on the results from Figure 4A and 4B, we next examined the combination effects of measles virus with 4EGI-1, a small-molecule inhibitor of translation initiation [17], quantitatively using Chou-Talalay methodology [18]. With the Chou-Talalay approach growth effects of MV-Edm, 4EGI-1, and the combination of MV-Edm and 4EGI-1 were measured in the panel of 4 MM cell lines in a 96 well plate format. Analysis was performed using Compusyn software and the combination indices (CI) determined (Table 1). For these studies and by definition, synergism (CI <1) is greater than an additive effect (CI =1) and antagonism is lower than an additive effect (CI >1). The combination effects of MV-CEA and 4EGI-1 for the 4 MM cell lines determined for the six different agent combinations per cell line yielded CI values indicating from slight to strong antagonism (C > 1) (Table 1). The average CI for the 6 different drug combinations for cell line H2373 was slightly antagonistic (1.12), for H2596 was moderately antagonistic (1.31), for H2461 was antagonistic (2.18) and for H513 was strongly antagonistic (8.31) [18]. These results, for each cell line indicating antagonism, also suggest that the strength of viral oncolysis is dependent upon active protein synthesis.

**Table 1:** Combination indices for the treatment of mesothelioma cell lines with measles virus (multiplicity of infection (MOI)) and 4EGI-1

H513		
**MV (MOI)**	**4EGI-1 (μM)**	**Combination Index**
1.0	25	1.99
1.0	50	4.53
1.0	75	9.76
2.0	25	4.95
2.0	50	9.02
2.0	75	19.6
**H2373**		
**MV (MOI)**	**4EGI-1 (μM)**	**Combination Index**
1.0	50	1.07
1.0	75	0.98
1.0	100	1.05
2.0	10	1.33
2.0	25	1.19
2.0	50	1.12
		
**H2461**		
**MV (MOI)**	**4EGI-1 (μM)**	**Combination Index**
0.25	10	2.45
0.25	25	3.89
0.25	50	1.82
0.5	10	1.52
0.5	25	1.74
0.5	50	1.69
**H2596**		
**MV (MOI)**	**4EGI-1 (μM)**	**Combination Index**
0.25	10	1.31
0.25	25	1.41
0.25	50	1.04
1.0	10	1.33
1.0	25	0.98
1.0	50	1.81

### Measles virus activity is enhanced by stimulation of host cell translation

**Figure 5:**
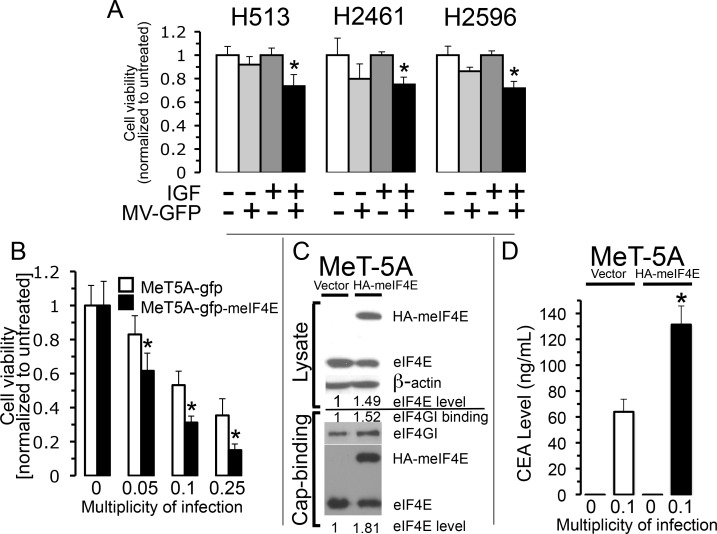
Stimulation of host cell translation enhances measles virus activity Activation of cap-dependent translation was induced by stimulation of MM cells with application of IGF-I (5 nM) followed by treatment with and without MV-GFP (MOI of 0.1) and viable cells counted 24 hours later by trypan exclusion. Cells were grown in serum free medium prior and after treatment. *Columns*, the mean of three independent determinations of cell viability normalized to untreated cells, *bars*, standard deviation. Asterisks denote a significant difference in cell survival (*p*<0.02) between IGF-I treated cells and the same cells infected and not infected with MV-GFP **(A)**. MeT-5A (mesothelial) cells that produce or don't produce mouse-eIF4E (meIF4E) were treated with increasing MOI of MV-CEA. Viable cells were counted 72 hours after treatment. Data represent the mean (+/− standard deviation) of six independent determinations of cell number normalized to untreated cells. Asterisks indicate a significant difference in cell viability (*p*<0.012) between cells producing and not producing meIF4E treated with the same MOI of MV-CEA **(B)**. Results from an examination of eIF4F integrity, employing a cap-affinity assay, for MeT-5A cells that produce or don't produce ectopic HA-tagged-meIF4E in cell lysates. *Top*, the band intensity levels for HA-meIF4E and eIF4E was determined in cell lysates derived from cells grown at steady-state. β-actin serves as a loading control. *Bottom*, levels of eIF4E, HA-meIF4E and eIF4GI eluted from 7-methyl-GTP-sepahrose resin. The amount of eIF4E plus meIF4E expressed in MeT-5A cells was compared to the amount of eIF4E in empty vector cells from both lysates and for proteins (HA-meIF4E, eIF4E and eIF4GI) eluted from the cap-analogue **(C)**. Band intensity levels were determined by using ImageJ. CEA level was assessed by collecting medium from MeT-5A cells that produce or don't produce meIF4E following treatment with or without MV-CEA (MOI 0.1). ELISA technique was used to examine the presence of CEA. Standard deviation is signified by error bars. Denoted by asterisk is the significant difference in CEA levels between MeT-5A cells that produce or don't produce meIF4E (*p*<0.005) **(D)**.

Results from three different methods to attenuate cap-mediated translation that lead to suppressed oncolytic activity of MV-Edm in mesothelioma were just revealed. To examine the role that enhanced protein translation has on the potency of MV oncolytic activity, two different approaches to stimulate cap-mediated translation were employed. Previous studies demonstrated that IGF-I stimulation of mesothelioma cells directly promotes activation of the eIF4F complex and thereby triggers enhanced cap-dependent translation [10]. To assess the impact that IGF-I stimulation has on the activation of MV oncolysis, three MM cell lines were left untreated or treated with IGF-I (5 nM), MV-GFP (MOI 0.1) or treated with both agents (Figure 5A) and cell viability measured 24 hours later. In all cell lines the cell viability was significantly reduced to a greater extent in the presence of IGF-I when treated with MV-GFP than in cells treated with MV-GFP but in the absence of IGF-I. The added efficacy of MV was likely due to elevated host cell translation machinery for progeny virion production, engendered by IGF-I stimulation.

In most examples the oncogenic potential of deranged eIF4F hyperactivity is connected to eIF4E activity, which is the rate-limiting element of the translation initiation complex [19, 20]. Moreover, in NIH-3T3 cells, ectopic overexpression of eIF4E was revealed to be oncogenic driving tumorigenesis [21]. In order to investigate the possibility that enforced overexpression of eIF4E would confer enhanced translation potency that would lead to improved cytotoxity by MV-Edm strain, MeT-5A cells were transfected with a vector either encoding or not encoding HA-tagged eIF4E from mouse (meIF4E) and then treated with MV-Edm at increasing MOIs (Figure 5). To confirm expression of meIF4E in MeT-5A cells and its impact upon cap-mediated translation immunoblot analysis was performed and the band intensities ascertained and normalized to their respective intensities found in the MeT-5A cells bearing empty vector (Figure 5C). It was revealed that the addition of meIF4E expression to the MeT-5A cells increased overall eIF4E levels 1.49 fold while enhancing the binding of eIF4GI and eIF4E in the cap-affinity assay by 1.52 and 1.81 fold, respectively, compared to cells lacking meIF4E. These results indicate elevated cap-meditated translation for MeT-5A producing ectopic meIF4E. MeT-5A cells either producing or not producing meIF4E was then treated with MV-CEA at the indicated MOI and assayed for cell viability 72 hours later. MeT-5A cells expressing ectopic meIF4E were significantly more sensitive to MV-Edm than cells not producing ectopic meIF4E at each MOI employed (Figure 5B). In order to confirm that the improved cytotoxicity from stimulated host cell cap-dependent translation is due to increased viral production, CEA levels in culture medium from cells producing and not producing meIF4E was measured. CEA marker emitted into the medium following MV-CEA infection of MeT-5A cells expressing ectopic meIF4E increased significantly compared to MV-CEA infected MeT-5A cells not expressing meIF4E (Figure 5D). This indicates that during stimulation of host cell translation MV-CEA replication is enhanced. Together, these data show that cells with hyperactivated cap-mediated translation induces enhanced oncolytic measles virus activity.

## DISCUSSION

The attenuated Edmonston vaccine strain of measles virus (MV-Edm) has become one of the promising replicating oncolytic viruses that are capable of infecting and killing tumor cells by proliferating in cancer tissues thereby magnifying their antineoplastic impact [2]. A number of factors control the proficiency of a virus to infect a cell. A mixture of permissiveness (viral replication) and susceptibility (viral entry) governs viral tropism for a host cell and is necessary for a virus to establish infection. MV-Edm oncolytic specificity has been shown to rely on the differential expression of the CD46 receptor [5], defects in the type I IFN response [22] and defects in antiviral innate immune response [23, 24]. In the present study, and in earlier investigations [22, 25], the CD46 receptor has revealed to be highly expressed in mesothelioma cells, and thus explains a large part of the susceptibility of infection. In the work presented here mesothelioma cells were shown not to be reliant on nectin-4 for viral entry. Most human malignancies exhibit increased levels of CD46 protein, and overexpression of regulatory membrane complement cofactor protein CD46 can assist in evading complement lytic activity for cancer cells [26]. While the elevated levels of CD46 can enhance the malignant phenotype in cancer it also engenders the tumor cells more sensitive to infection by MV-Edm potentiating cell killing.

A basic function following viral infection remains redirection of normal cellular metabolism towards processes favoring viral replication. For wild-type HL strain of MV it is suggested that the MV-N protein shuts off host translation in MV-infected cells through protein-protein interactions with host cell eukaryotic initiation factor 3 (eIF3) [27]. It is believed that this same procedure works for MV-Edm strain. This mechanism for viral permissiveness is critical and functions through subversion of cap-mediated translation towards production of critical viral proteins. This may be particularly important in cancer cells where often the activation of the eIF4F complex is associated with production of critical proteins needed for both maintaining the malignant phenotype and blocking apoptosis.

Although eukaryotic translation is mediated through both cap-mediated mechanisms as well as IRES, the default translational complex in most cancer cells appears to be eIF4F directed [28]. Based on the current results, the permissiveness of mesothelioma cells to oncolytic viral therapy appears to depend in a large part to ongoing presence of hyperactivation of cap-mediated translation. In the studies presented here, the stimulation of cap-mediated translation by either IGF-I exposure or forced expression of eIF4E induced enhanced measles virus oncolytic activity. And the opposite is true as well. Repression of cap-dependent translation by forcing mesothelioma cells to produce an activated repressor protein or with the treatment of mesothelioma with agents [4EASO, 4EGI-1] that suppress host cell translation decreases the potency of oncolytic viral efficacy. This argues strongly that transformed mesothelial cells will be preferentially susceptible to viral oncolysis imparting selectivity towards the malignant phenotype for this therapy. This is particularly important in view of the deleterious effects of infection of normal host cells by wild-type MV.

In addition, selectivity for measles viral therapy in mesothelioma is likely mediated through over-expression of the CD46 receptor protein when compared to non-transformed mesothelial cells. Although the vast majority of the population is immunized to measles, there remains a benefit to developing a viral therapy that demonstrates selectivity towards cancer cells over non-transformed host cells. In this regards, both the preferential expression of the CD46 receptor and activation of the eIF4F complex in mesothelioma cells are important in defining measles virotherapy as a safe and targeted treatment in mesothelioma. In ovarian cancer, like mesothelioma, the CD46 receptor is also over-expressed [29] and cap-mediated translation is aberrantly stimulated [8]. Given these attributes a phase I/II trial of MV-Edm in patients with recurrent ovarian cancer was launched. In this study intraperitoneal treatment was well tolerated and correlated with favorable overall survival. [30]. The result of a Phase 1 trial of measles virus therapy in pleural mesothelioma has not finalized but looks promising as well (ClinicalTrials.gov identifier NCT01503177).

A number of targeted cancer treatments in both clinical use and development target pathways that impact protein translation through mTOR inhibition [8]. It is important to note that a primary target of mTOR is activation of protein translation via phosphorylation and consequent deactivation of the 4EBP1 repressor protein. It is reasonable to view with caution combining viral oncolytic therapies with treatments that target mTOR or the eIF4F complex. Based on the findings in the current study, it would be expected that such a strategy would not be additive in its cytotoxicity but more likely antagonistic.

Mesothelioma remains a lethal cancer with few effective therapies. Given the intracavitary nature of the disease and high expression of CD46, measles therapy remains an interesting and promising approach. A primary characteristic of mesothelioma cells, and of most transformed cells, remains the activation of eIF4F-mediated translation. It is apparent that this phenotype renders mesothelioma susceptible to this oncolytic therapy and deserving of continued clinical development.

## MATERIALS AND METHODS

### Cell lines, cell culture and viruses

The medium for MM cell lines, H513, H2373, H2461 and H2596 (American Tissue Culture Collection (ATCC), Manassas, VA) was RPMI 1640 (Gibco, Life Technologies, Waltham, MA) containing 10% newborn calf serum (Sigma-Aldrich, St. Louis, MO). LP9 cells, non-transformed human mesothelial cells (Coriell Institute for Medical Research, Camden, NJ), were propagated in a medium containing a 1:1 ratio of M199 and MCDB10 basal medium (Sigma-Aldrich) supplemented with 15% newborn calf serum [not heat inactivated], 2 mM glutamine, 10 ng/mL epidermal growth factor (EGF) and 0.4 μg/mL hydrocortisone. MeT-5A (ATCC) cells (non-transformed, SV40 immortalized mesothelial cells) were grown in M199 containing Earle's BSS, 0.75 mM L-glutamine, 1.25 g/L sodium bicarbonate supplemented with 3.3 nM EGF, 400 nM hydrocortisone, 870 nM insulin, 20 mM HEPES, trace elements and 10% calf serum. All cells were maintained at 37°C in 5% CO_2_. The authenticity of these cell lines were verified by short tandem repeat analysis (Johns Hopkins Cell Authentication Facility).

Recombinant MV-Edmonston vaccine strain (MV-Edm) bearing the gene that encodes green fluorescent protein (MV-GFP) or encoding the gene for the soluble extracellular domain of human CEA (carcinoembryonic antigen) (MV-CEA) were constructed and propagated as described previously [31]. The titer of viral stocks was determined by 50% end-point dilution assays [(TCID_50_)/mL] performed on Vero cells [32].

### Cell lysate preparation

The cells were removed from the plates by scraping after washing once with PBS (phosphate buffered saline), after which cells were collected by centrifugation (14K rpm, 14 seconds), washed again with ice cold PBS followed by another round of centrifugation and resuspended in a volume of TNESV lysis buffer (50 mM Tris-HCl, pH 7.4; 1 % NP-40; 2 mM EDTA, pH 8.0; 0.1 M NaCl) containing protease (Roche, Penzberg, Germany) and phosphatase inhibitors (Sigma-Aldrich). In instances that cell lysates were used for cap-affinity assays, cells were resuspended in a volume of freeze-thaw lysis buffer (50 mM Tris-HCl pH 7.5, 150 mM NaCl, 50 mM NaF, 1 mM EDTA, 10 mM tetrasodium pyrophosphate) containing the same protease and phosphatase inhibitors followed by three freeze-thaw cycles (15 min at −80°C, 2 minutes at 37°C). Protein lysate concentration was determined by Bradford assay (Bio-Rad, Hercules, CA) and stored at −80°C.

### Cell transfection

MeT-5A, SV40 immortalized mesothelial cells, were transfected with previously described [33] pMSCV-M1GR1 constructs, either encoding or not encoding HA-tagged translational regulator eIF4E from mouse (meIF4E), linked to green fluorescent protein (GFP) using lipofectamine 2000 (Invitrogen, Life Technologies, Waltham, MA) following manufacturer's instruction. GFP-positive cells were sorted at 488 nm laser emission on a fluorescence-activated cell sorting (FACS) DiVA (BD Biosciences, Franklin Lakes, NJ) with a 530/528 optical filter followed by immunoblot analysis of collected cells to assess expression of HA-tagged meIF4E protein.

Cell line H2596 was transfected with pACTAG-2 (empty vector) or pACTAG *neo*/HA-4EBP1^A37/A46^ to generate cell lines H2596 empty vector and H2596 4EBP1^A37/A46^ utilizing G418 (Media Tech Inc., Manassas, VA, catalog number 30234CR) (*neo* gene) resistance as described [10].

### *In vitro* cap-affinity assay

The strength of cap-mediated complex formation was measured as before [10]. Lysate (300 μg) from MeT-5A cells that either do or do not produce exongeneous HA-tagged mouse eIF4E were diluted in 300 μL freeze-thaw lysis buffer and combined with 50 μl of a 50% slurry of 7-methyl GTP-Sepharose^TM^4B (Amersham Biosciences, Piscataway, NJ) and incubated while mixing for two hours at 4°C. Freeze-thaw lysis buffer containing 100 μM 7-methylguanosine 5′-triphosphate (Sigma-Aldrich) was employed, along with heating at 95°C for 5 minutes, to elute the captured binding partners, eIF4E and eIF4GI from the 7-methyl GTP-Sepharose^TM^4B beads. The eluted samples were then prepared for immunoblot analysis. The density of protein bands was determined by using ImageJ (National Institutes of Health, Bethesda, MD), a public domain Java image processing program. The protein bands for eIF4E and eIF4GI were normalized to their respective densities found in the MeT-5A empty vector sample.

### Immunoblot analysis

Protein samples for the cap-affinity assay were separated by 8-15% gradient SDS-PAGE (polyacrylamide gel electrophoresis). Ten or twelve percent SDS-PAGE was used for PARP and eIF4E proteins, respectively. Following protein transfer to PVDF (Amersham Biosciences) the membranes were blocked in 5% non-fat dry milk for 1 hour at room temperature in Tris-buffered saline-Tween (TBST: 0.15 M NaCl; 0.01 M Tris-HCl, pH 7.6; 0.05% Tween 20). The membranes were then incubated for 1 hour at ambient temperature or overnight at 4°C with the chosen primary antibody. The primary antibodies employed were rabbit α-eIF4E (catalog number 9742), rabbit α-PARP (catalog number 9542), α-SV40 large T antigen (catalog number 15729) all from Cell Signaling Technology (Danvers, MA) at a 1:1000 dilution, mouse α-β-actin (Sigma-Aldrich, catalog number A1978) at a 1:10,000 dilution and rabbit α-eIF4GI (kindly provided by Nahum Sonenberg, McGill University Montreal, QC, Canada) at a 1:2500 dilution. Preceding and following incubation with the appropriate horseradish peroxidase labeled secondary antibodies, the blots were washed three times for 5 minutes in TBST and detection was then performed utilizing ECL Plus Western Blotting System (Amersham Biosciences) to visualize the bands of interest.

### Viral infection alone and combined with drug treatment

For the results in Figure 1A, each cell line was seeded in 6 well plates at 1 × 10^5^ cells per well, incubated overnight and treated or not treated with MV-GFP at an MOI of 1.0. At 48 hours after infection cells were photographed employing light and fluorescence microscopy. For the results in Figure 1B and 1C, MeT-5A and MM cells were seeded at 1 × 10^5^ cells per well in 6 well plates. Following overnight incubation the cells were washed with Opti-MEM (Gibco, Life Technologies) and then infected with MV-CEA at different multiplicities of infection (MOI) in 0.4 mL of Opti-MEM for 2 h at 37°C. Complete medium for each cell line was then added to the wells for the duration of the treatment.

Insulin-like growth factor (IGF-I) (ABR Affinity Bioreagents, Golden, CO), stimulation (5 nM) was carried out on 1 × 10^5^ cells per well following overnight incubation. After overnight IGF-I stimulation cells were treated or not treated with MV-GFP at an MOI of 0.1. Subsequent to the MV-GFP infection, IGF-I at 5 nM was maintained in the appropriate wells. At 24 hours after infection cells were counted.

Second-generation antisense oligonucleotides (ASOs), containing a phosphorothioate backbone, were provided by Eli Lilly and Company (Indianapolis, Indiana) and were 20 nucleotides in length [34]. 4EASO has the sequence, 5′-TGTCATATTCCTGGATCCTT-3′, where the underlined nucleotides are 2′-methoxyethyl-modified (MOE). It was designed to target the 3′ untranslated region of human *eIF4E* mRNA. The mismatch ASO (mmASO) control has the sequence 5′-TCTTATGTTTCCGAACCGTT-3′ containing the same base composition as 4EASO. Oligofectamine (Invitrogen, Life Technologies) was employed for ASO transfection as described previously [15]. Following ASO transfection cells were then infected or not infected with MV-CEA as described above with subsequent cell harvest at 48 hours or counted at 72 hours at the indicated MOIs. All cells for proliferation studies were counted by trypan blue exclusion employing a hemacytometer. Cell survival is expressed as cell number normalized to untreated cells. Experiments were performed in triplicate.

For experiments employed to determine combination indices (CI), 5000 cells per well were seeded in the presence of 1% serum in each well of 96-well plates. Following overnight incubation, the cells were either left untreated or treated with the indicated MOI of MV-CEA for 2 hours. The cells were then treated with 4EGI-1 (ChemBridge, San Diego, CA, ID:5154300) at the desired concentrations and medium was added to produce a final serum concentration of 10%. The cells were next incubated at 37°C for 72 hours. Cell viability was measured by using Cell Counting Kit-8 (Dojindo Molecular Technologies, Rockville, MD). Experiments were performed in triplicate and cell viability values normalized to untreated cells. The degree of cooperation between 4EGI-1 and MV-CEA subsequent to treatment was determined using the Chou-Talalay method employing CompuSyn software. The resulting combination index (CI) quantitatively depicts synergism (CI<1), additive effect (CI=1) and antagonism (CI>1) [18].

### CEA level determination

MeT5-A and MM cells were treated with the indicated MOI of MV-CEA. Seventy-two hours later cell medium was collected from infected and uninfected cells and analyzed for CEA concentration. The CEA levels were measured using CEA ELISA kit (Bio-Quant, Inc., San Diego, CA) following manufacturer's instructions.

### Annexin V measurement

Phosphatidylserine externalization was determined as an indicator of apoptosis utilizing Guava Nexin Reagent and the Guava EasyCyte flow cytometer (EMD Millipore, Guava Technologies, Billerica, MA). Cells (1 × 10^5^ per well) were infected or not infected with MV-GFP at an MOI of 1.0 as outlined above in six well plates for 48 hours. The Guava Nexin reagent was added to harvested cells and incubated in the dark for 20 minutes at room temperature. Samples were analyzed using flow cytometry per manufacture's recommendation. Results are expressed as the percentage of gated cells that are positive for Annexin V staining. A total of 5000 events were analyzed per sample.

### Assessment of CD46 and nectin-4 levels

Cells were grown to ∼70-80% confluency, harvested, washed in 1X PBS containing 1% bovine serum albumin (PBS-BSA), centrifuged and resuspended in PBS-BSA. Cells were then incubated for 30 minutes at 4°C with phycoerythrin (PE) CD46 antibody (Abcam, Cambridge Science Park, Cambridge, UK), anti-nectin-4-PE (R & D Systems, Minneapolis, MN, catalog number FAB2659) or control isotype-matched antibodies (Invitrogen, Life Technologies), centrifuged and resuspended in PBS-BSA. Cells were analyzed for CD46 or nectin-4 level on a Becton Dickinson (Franklin Lakes, NJ) FACSCalibur cytometer. Analysis was done using FlowJo (Ashland, OR) software.

### Blocking antibodies against CD46 and nectin-4

H513 cells (1 × 10^4^ per well) were seeded onto 96-well plates in Opti-MEM. The following day cells were treated with isotype IgG (R & D Systems, catalog number AB-108-C), or nectin-4 IgG (R & D Systems, catalog number AF2659) or CD46 IgG (Santa Cruz, Dallas TX, catalog number sc-5267-clone m177) or both CD46 and nectin-4 antibodies. All antibody concentrations are 100 μg/mL. One hour following antibody additions, MV-GFP was added to the wells at an MOI of 1.0. After an infection of 2 hours, complete medium was added to the cells. 48 hours later cells were analyzed by fluorescence and light microscopy. In parallel, fluorescence of triplicate sets of treated cells was measured employing a 96-well plate reader. Untreated and MV-GFP infected cells without blocking antibodies were used as negative and positive controls, respectively.
